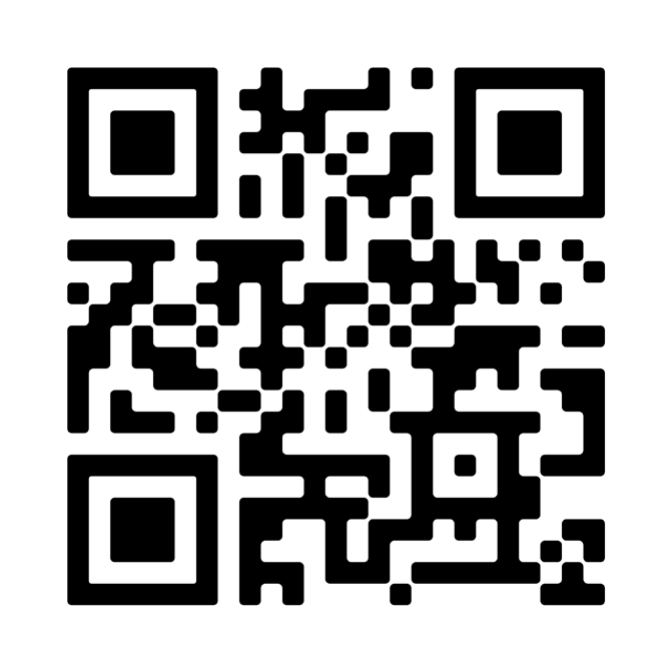# Vaginal Leiomyomas - Safe Steps for Laparoscopic removal: feasibility from 3 case reports

**DOI:** 10.52054/FVVO.15.2.067

**Published:** 2023-06-30

**Authors:** E Giovannopoulou, S Kogeorgos, A Lazaridis, G Pistofidis

**Affiliations:** Tertiary Referral Center of Gynecological Laparoscopy, Lefkos Stavros Hospital, Athens, Attiki, Greece, PC 11528

**Keywords:** Gynaecologic laparoscopy, interventional endoscopy, surgical education

## Abstract

**Background:**

Leiomyomas are a common pathology in reproductive-aged women. However, they rarely originate from extrauterine sites. Vaginal leiomyomas constitute a challenging diagnosis, regarding their surgical treatment. Despite the well- established advantages of laparoscopic myomectomy, the efficacy and feasibility of a total laparoscopic approach for such cases has not been yet investigated.

**Objectives:**

To describe step-by-step the laparoscopic technique for vaginal leiomyoma removal (narrated video presentation) and demonstrate the outcomes of a small series managed at our institution.

**Patients:**

Three patients diagnosed with symptomatic vaginal leiomyomas that presented to our laparoscopic department. Patients aged 29, 35 and 47 years with BMI 20.6 kg/m2, 19.5kg/m2 and 30.1 kg/m2, respectively.

**Results:**

Total laparoscopic excision of the vaginal leiomyomas was successful in all three cases without conversion to laparotomy. The technique is demonstrated in a step-by-step video narration. There were no major complications. Average operative time was 146.25 min (range 90- 190 min) and intraoperative blood loss was 120 ml (range 20-300ml). Fertility was preserved in all patients.

**Conclusion:**

Laparoscopy is a feasible technique to approach vaginal masses. Further studies are needed to assess safety and efficacy of the laparoscopic technique in such cases.

## Learning Objective

In-depth laparoscopic pelvic anatomy and safe techniques are demonstrated in this surgical video. The following steps are considered important learning points: visualisation of the ipsilateral ureter; lateral retroperitoneal approach to enter the prevesical, right medial paravesical and paravaginal spaces; mobilisation of the bladder from the anterior abdominal wall to expose the anatomical relation of the leiomyoma with the trigone and the urethra at the base of the bladder and restoration of the anterolateral vaginal wall defect with interrupted absorbable sutures.

## Introduction

Uterine leiomyomas are the most common benign neoplasms of the genital tract in premenopausal women (Stewart, 2001; De La Cruz and Buchanan, 2017). Their predominant location is in the uterus, but extrauterine locations have been described (Fasih et al., 2008). The genitourinary tract including the ovaries, vagina, vulva, bladder, and urethra have all been described as potential extrauterine sites (Fasih et al., 2008). Vaginal leiomyomas develop on the anterior vaginal wall (69.5%), and less frequently on the posterior (17%) and lateral (13.5%) walls (Braga et al., 2020; Çobanoǧlu et al., 1996).

Several surgical techniques have been described for the excision of uterine fibroids including laparotomy, hybrid and total laparoscopic procedures (Faivre et al., 2010; Holub et al., 2005; Jin et al., 2009). Laparoscopic myomectomy has well-established advantages with reduced blood loss, faster postoperative rehabilitation with less pain compared to laparotomy (Jin et al., 2009). Vaginal approach of uterine fibroids has been described as an alternative to minimise surgical trauma associated with laparotomy and reduce longer operative times associated with laparoscopy (Jin et al., 2009).

However, in the subgroup of patients with vaginal leiomyomas, the literature lacks sufficient data on the potential role of minimally invasive procedures. The scope of the present study is to evaluate the feasibility and the operative parameters of a total laparoscopic approach for the excision of anterior vaginal leiomyomas by presenting a series of successful cases conducted in our department.

## Patients and methods

Three patients that presented with variable symptoms of pelvic pain, dyspareunia, or void dysfunction to our hospital. The diagnosis of an anterior vaginal leiomyoma was made in all three cases preoperatively. All patients underwent a laparoscopic procedure for fibroid removal by the same laparoscopic surgical team.

After careful pre-operative evaluation, the laparoscopic approach was preferred to the vaginal route as it offers superior exposure of the surgical field coupled with detailed demonstration of anatomical landmarks. Specifically, the leiomyomas paravesical topography, its relationships to the urethra, bladder, ureter as well as the parametrial and paracervical vasculature can all be better appreciated at laparoscopy.

The decision to proceed with laparoscopy rather than a vaginal approach was made after a thorough preoperative evaluation of risk factors for conversion to laparotomy such as uterine size, leiomyoma topography (anatomical relationship to the urethra, the bladder, and the ureter) or suspected pelvic adhesions from previous surgery.

All patients provided informed written consent. The study was approved by the Institutional Review Board (IRB) of the hospital.

## Results

**Figure 1:**
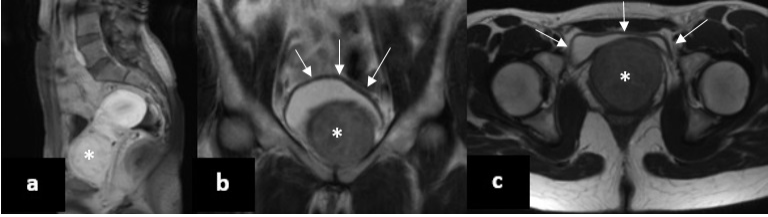
(# Case 1) MRI scan showing the exact location of the leiomyoma at the anterior vaginal wall at (a) sagittal (T1- weighted), (b) frontal and (c) transverse planes (T2-weighted). The leiomyoma is indicated with an asterisk (*), while the circumference of the urinary bladder with multiple arrows.

**Figure 2:**
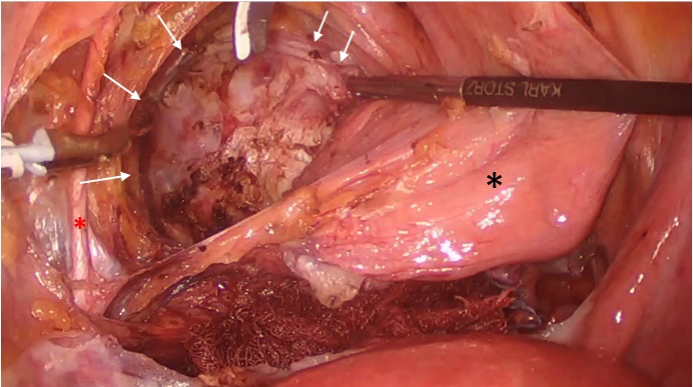
(# Case 1) Laparoscopic view of the left anterior wall leiomyoma (indicated with multiple arrows) through the left median paravesical and paravaginal spaces, after adequate mobilisation of the urinary bladder (black asterisk *). The obliterated left umbilical artery is visulaized at the left (red asterisk *). A gauze has been placed at the vesico-uterine fold for hemostasis.

**Figure 3:**
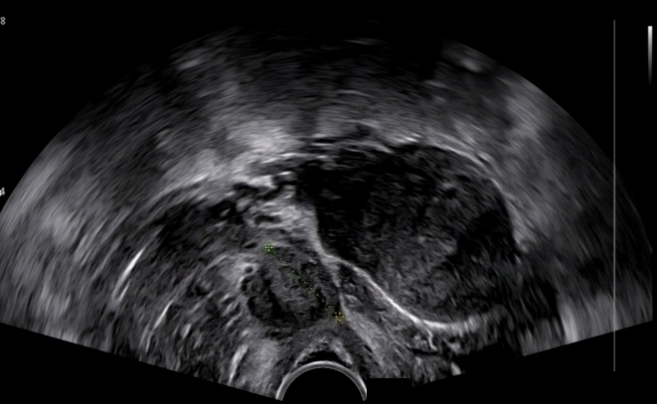
(# Case 2). Ultrasound scan depicting the 4cm leiomyoma at the anterior vaginal wall (transverse plane).

**Figure 4:**
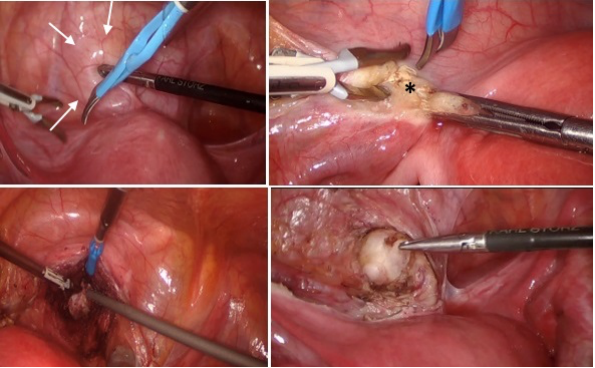
(# Case 2) Step-by-step the laparoscopic approach of the 4cm vaginal leiomyoma, indicated with multiple arrows. Left round ligament (with an asterisk *) is dissected to approach the mass through the retroperitoneum.

**Figure 5:**
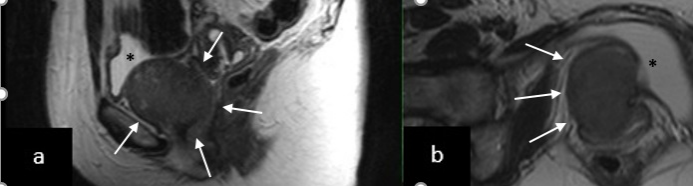
(# Case 3) MRI image of the 6cm vaginal leiomyoma occupying the upper portion of the anterior vaginal wall at (a) sagittal and (b) transverse planes (T2- weighted). The mass (multiple arrows) applies external pressure to the urinary bladder (indicated with an asterisk).

Patient’s characteristics and measured outcomes are summarised at Table I. Pre-operative imaging and intraoperative figures are also available for each case (Figure 1-5).

**Table I:** Basic demographic characteristics of the patients, characteristics of the leiomyomas and operative outcomes (intraoperative, postoperative).

Case	Age (Years)	BMI (kg/m^2^)	Leiomyoma location	Maximal diameter (cm)	Operative time (min)	Estimated blood loss (ml)	Intraoperative complications	Postoperative complications	Hospital stay (days)
#1	38	19.5	Medial paravesical space	8	190	300	Venous Bleeding	None	2
#2	29	20.6	Medial paravesical space	4	90	20	None	None	1
#3	47	30.1	Medial paravesical space	6	160	40	None	None	1

## Video demonstration

A 47 –year old, otherwise healthy woman was referred to our department with voiding dysfunction. The patient was experiencing intermittent urinary flow and recurrent episodes of urinary retention during the preceding 12 months. BMI was 30.1 kg/m^2^. In the past, she underwent a diagnostic laparoscopy procedure for subfertility, which was unremarkable, and an operative hysteroscopy to remove an endometrial polyp. At presentation, a solid mass protruding into the anterior vaginal fornix was palpated during bimanual examination. Initial sonographic evaluation revealed a six cm lesion at the anterior vaginal wall that applied external pressure to the urinary bladder and the urethra. A pelvic MRI scan revealed a mass resembling a myoma that occupied the space between the upper third of the anterior vaginal wall, lying in close proximity to the urethra and bladder neck. The mass was adjacent to the distal segment of the right ureter without MRI features indicative of extrinsic compression, such as ipsilateral hydroureter or hydronephrosis (Figure 5). Two months prior to our laparoscopy the patient had undergone laparotomy in another institution for vaginal mass excision, which failed to reveal the myoma located at the lower genital tract. She was subsequently referred to our centre for laparoscopic management. During our procedure, cystoscopy was performed, which showed external pressure of the inferior bladder wall and the urethra. The mass was safely approached and completely excised by laparoscopy. The Valtchev uterine manipulator was utilised in the procedure. The operative time was 160 min and blood loss was 40 ml. No complications were recorded. The laparoscopic procedure is presented on video.

## Discussion

Surgical excision of symptomatic vaginal leiomyomas is the proposed treatment (Braga et al., 2020; Gottwald et al., 2003). To date, 90% of cases have used a vaginal approach (Braga et al., 2020). The remaining 10% were managed via the abdominal route with laparotomy (Braga et al., 2020). A combined abdominal-perineal approach was proposed for larger masses (Chakrabarti et al., 2011).

A recent systematic review of the literature on this subject commented on the surgical management of vaginal fibroids at the anterior compartment (Braga et al., 2020). Eighty-six related cases were included and none of them was managed with a laparoscopic approach. According to our literature review, no additional cases have been added in the literature since then.

Few attempts to approach vaginal leiomyomas laparoscopically are presented in the literature (Braga et al., 2020; Iyer and Al-Inizi, 2013; Manoucheri and Einarsson, 2013; Zhang et al., 2020). In all these cases except one, a laparoscopic approach was used either to guide vaginal excision or during total laparoscopic hysterectomy.

In 2013, Iyer and Al-Inizi (2013) described a case of laparoscopically guided vaginal excision of a vaginal leiomyoma located laterally at the posterior fornix. The mass measured 3.6 cm and was completely dissected by a vertical incision on the lateral vaginal wall. Laparoscopic view was used to prevent injury in neighbouring structures and, especially the right ureter.

During the same period, Manoucheri and Einarsson (2013) conducted a complete laparoscopic removal of a vaginal mass, later proved to be a leiomyoma. In this case, the patient underwent total laparoscopic hysterectomy for uterine fibroids and the extrauterine leiomyoma was located near the vaginal cuff.

In July 2020, Zhang, and colleagues reported a total laparoscopic technique for the removal of a 5-cm vaginal fibroid at the upper vagina of a 34-year-old woman. The procedure lasted 95 minutes and there were no surgical complications. The woman was followed for the next 20 months; she subsequently became pregnant and had an uneventful vaginal delivery (Zhang et al., 2020).

Here, we demonstrate a total laparoscopic approach for the removal of vaginal leiomyomas, based on the proven benefits of laparoscopic myomectomy over laparotomy (Jin et al., 2009). Data on the optimal approach for the excision of vaginal leiomyomas are sparse due to the rarity of this pathology. Based on our experience, laparoscopy offers advantages over both laparotomy and the vaginal approach. The vaginal approach may be faster but entails risks of accidental bleeding and injury to adjacent organs due to limited visibility. Laparoscopy offers superior safety due to increased visibility of the anatomical structures. However, this hypothesis needs to be supported by subsequent randomised controlled studies.

Concerning the limitations of laparoscopy in such cases, operation at this level requires advanced operational skills and good anatomical knowledge of the lower pelvis and the perineal region. Key factors for such cases include meticulous haemostasis, opening up anatomical spaces, identification of structures and complete exposure of the myomas.

## Conclusions

Laparoscopy is a technically feasible and minimally invasive approach for vaginal leiomyomas. We feel that it may benefit specific groups of patients especially in the context of fertility preservation, however randomised-controlled trials are needed to conclude on the optimal surgical technique for such cases.

## Video scan (read QR)

https://vimeo.com/812401875/517bc960f3?share=copy

**Figure:**